# Zinc supplementation improves body weight management, inflammatory biomarkers and insulin resistance in individuals with obesity: a randomized, placebo-controlled, double-blind trial

**DOI:** 10.1186/s13098-019-0497-8

**Published:** 2019-12-02

**Authors:** Hoda Khorsandi, Omid Nikpayam, Reyhaneh Yousefi, Maryam Parandoosh, Nima Hosseinzadeh, Atoosa Saidpour, Arman Ghorbani

**Affiliations:** 1 Department of Clinical Nutrition & Dietetics, National Nutrition and Food Technology Research Institute, Faculty of Nutrition Sciences and Food Technology, Shahid Beheshti University of Medical Sciences, 46, West Arghavan St., Farahzadi Blvd., Shahrak Qods, P.O. Box 19395-4741, Tehran, Islamic Republic of Iran; 2 Student Research Committee, Nutrition Research Center, Tabriz University of Medical Sciences, Tabriz, Iran; 3 Department of Clinical Nutrition, Faculty of Nutrition and Food Science, Tabriz University of Medical Sciences, Tabriz, Iran; 4 Faculty of Biostatistics, Shahid Beheshti University of Medical Sciences, Tehran, Islamic Republic of Iran; 5 Department of Cellular and Molecular Nutrition, Faculty of Nutrition Science and Food Technology, National Nutrition and Food Technology Research Institute, Shahid Beheshti University of Medical Sciences, Tehran, Iran

**Keywords:** Zinc supplement, Obesity, Anthropometric measurements, *hs*-CRP, Insulin resistance

## Abstract

**Background:**

The present study was designed to determine whether zinc supplementation would increase the effects of restricted calorie diet (RCD) on obesity.

**Methods and materials:**

A randomized, double-blind clinical trial was performed on 40 obese subjects who were randomly assigned to receive zinc supplements (30 mg/day) or placebo for a period of 15-weeks. Both groups were under a restricted calorie diet (~ 300 kcal lower than the estimated energy requirement). Anthropometric measurements, biochemical markers, appetite, and dietary intakes were determined during the study period.

**Results:**

The reductions of body weight, body mass index, waist circumference, and hip circumference were significantly higher in the zinc group compared to the placebo group (*P *= 0.032, 0.025, 0.003, and 0.0001, respectively). Lower levels of high sensitivity C-reactive protein, apelin, homeostatic model assessment of insulin resistance (HOMA-IR), and appetite score were observed in the zinc group in comparison with the placebo group (*P *= 0.0001, 0.001, 0.031 and 0.001 respectively).

**Conclusion:**

This study indicates that Zn supplementation with a restricted calorie diet has favorable effects in reducing anthropometric measurements, inflammatory markers, insulin resistance and appetite in individuals with obesity, and may play an effective role in the treatment of obesity.

*Trial registration* This clinical trial was registered at clinicaltrials.gov at the U.S. National Library of Medicine (NCT02516475).

## Background

As the etiology of obesity is complex [1], current interventions for weight management are only modestly successful [2]. Restricted calorie diets (RCD) are playing a fundamental role in prevention and treatment of obesity [3, 4]; but these diets often result in micronutrient deficiencies [5]. Furthermore, obesity and obesity-related inflammation are related to abnormal micronutrient status [5–7]. Among these micronutrients, zinc (Zn) deficiency is a common problem in obese individuals [8–10]. Furthermore, Zn has been reported as limiting nutrients in RDCs [11]. Previous studies have also demonstrated that plasma Zn level and dietary intake of Zn are insufficient in obese individuals [12–14]. So, it seems that further weight gain or development of obesity-related disorders may occur if the Zn deficiency is not corrected [15]. Payahoo et al. [16] also showed that daily intake of 30 mg Zn gluconate for 1 month decreased significantly body weight and body mass index (BMI). Two key assumptions about the possible mechanisms for the effects of Zn supplementation on weight loss are including appetite regulation [17] and improving insulin resistance (IR) [18, 19]. Another important aspect which worth to notice is the beneficial effects of dietary intake of Zn and plasma Zn level on inflammatory status [20, 21]. Zn has shown possible anti-inflammatory effects through cytokine signaling pathways [22] and the attenuation of plasma levels of Interleukin-6 (IL-6), tumor necrosis factor-α (TNF-α) and C-reactive protein (CRP) [23, 24]. Moreover, a growing body of literature has demonstrated that these inflammatory markers are directly or indirectly correlated with obesity-related IR through blocking the insulin signaling receptors activation in pancreatic β-cells [25]. More recently, apelin is also proposed as an adipokine mediator which might have an adaptive response to prevent chronic inflammation associated with obesity [26, 27]. Previous reports also imply that higher apelin levels are associated with both insulin resistance and chronic inflammation in individuals with obesity [28]. So, based on previous studies, low Zn concentration and high level of inflammatory markers maybe correlated to high BMI [29, 30], and it seems reasonable to assume that Zn supplementation may have favorable effects on weight loss or reversing obesity-related comorbidities such as IR. Therefore, this study was designed to evaluate the effects of daily intake of 30 mg Zn supplement along with RCD on anthropometric measurements, appetite, IR, and serum levels of inflammatory markers, apelin, and neuropeptide Y (NPY), in obese individuals.

## Materials and methods

### Study design and participants

This double-blind randomized clinical trial was conducted from December 2015 to April 2016. In order to detect a difference of 4.5 kg/m^2^ in the BMI and with respect to a pooled standard deviation of 26.21 kg/m^2^, obtaining from the study by Payahoo et al. [16], the sample size was calculated 20 subjects for each group. In this two-arm parallel study with two-tailed testing, a power (1–β) of 80% and α = 0.05 was used. Fifty healthy adults (men and women) with obesity and BMI more than 30 kg/m^2^ in the age range of 18–45 years were selected using convenience sampling from the Specialized Clinic of Nutrition & Diet Therapy located at the Faculty of Nutrition Sciences and Food Technology of Shahid Beheshti University of Medical Sciences in Tehran, Iran. In our study, exclusion criteria were the presence of pregnancy or lactation, chronic kidney or hepatic disease, autoimmune and infectious disease, chronic inflammatory diseases, recent surgery, smoking, having weight loss diets in the last 2 months, the use of Zn, calcium, or iron supplements in the last 2 months, and taking anticoagulant drugs, lipid-lowering or beta-blocker drugs. The primary outcomes were anthropometric measurements, and secondary outcome were appetite score, serum levels of inflammatory markers, apelin, NPY, glucose, Zn and insulin, and IR. The study protocol was approved by the Ethics Committee of the National Nutrition and Food Technology Research Institute of Iran (IR.SBMU.nntri.Rec.1394.407). The study was in adherence with the Declaration of Helsinki. Written informed consent was obtained from all subjects before initiating the study. This clinical trial was registered at clinicaltrials.gov at the U.S. National Library of Medicine (NCT02516475).

### Randomization

The subjects were randomly allocated to either a Zn or placebo group by block randomization. A trained dietitian completed the block randomization with a block size of 4 and possible balanced combinations with 2 P (placebo) and 2 Z (Zn supplement) subjects, calculated as 6 blocks (ZZPP, PZPZ, PZZP, ZPZP, PPZZ, ZPPZ). Then, blocks were randomly chosen, using a simple random sampling method to determine the assignment of all the participants into the groups.

### Intervention

During this study, subjects in the Zn group received 30 mg zinc sulfate as 1 capsule (between meals) while those in the placebo group received corresponding placebo capsules containing starch (also between meals). All capsules were produced by Dineh Iran Company, Tehran, Iran. According to the literature, zinc supplement is safe at a dose of 30 mg/day [31, 32]. Blinding was performed by a trained dietician, and the patients and researchers were kept blinded to the allocation. In addition, subjects in both Zn and placebo groups received a restricted calorie diet (RCD) with ~ 300 kcal lower than the estimated energy requirement based on the Mifflin-St Jeor equation in order to reduce their weight about 1 kg per month, and this RCD contained ~ 55% carbohydrate, ~ 15% protein and ~ 30% fat [33]. Adherence to the diet was monthly assessed by a registered dietitian. Participants were followed twice a month via telephone calls in order to ensure their compliance and were asked to maintain their usual physical activity level. They were also asked to return the remaining capsules, and based on the number of returned capsules by each subject and adherence to the diet, their degree of compliance was determined and the data of individuals with the degree of compliance more than 90% were analyzed at the end of the study.

### Dietary intakes and appetite assessments

Dietary intakes of participations were assessed using a 3-day dietary recall (2 weekdays and

1 weekend day) at baseline and at the end of week 15. Individuals’ diets were analyzed by Nutritionist IV software (N Squared Computing, San Bruno, CA, USA). Basal metabolic rate (BMR) was calculated based on Mifflin and St Jeor et al. [34]. Underreporting was defined as a ratio reported energy intake by 3-day dietary recall/BMR < 1.1 [35]. Simplified nutritional appetite questionnaire (SNAQ), a valid 4-item questionnaire recommended for clinical purposes [36], were used to assess the appetite at baseline and week 15. The SNAQ items were as follows: #1, Appetite; #2, Feeling full; #3, Food tastes; #4, Feeling hunger, and the sum of the 4 items scores constitutes the total SNAQ score which ranges from 4 to 20. The total score of 4 to 14 and 15 to 20 indicates low and normal appetite, respectively [36].

### Anthropometric assessments

Weight was measured with minimum clothes and without shoes using a calibrated scale (Seca, CA, USA) and precision of 100 g. Height was measured using a wall-mounted stadiometer with the precision of 0.5 cm. Hip and waist circumference were also measured using an inflexible tapeline with the precision of 0.5 cm, in the narrowest circumference below the rib cage and above the umbilicus and the largest circumference between the waist and knees, respectively [37]. BMI was calculated as the ratio of weight (kg)/height^2^ (m^2^). Anthropometric parameters were measured at baseline and at the end of weeks 7 and 15.

### Physical activity assessment

Physical activity level was estimated using a valid and reliable physical activity questionnaire [38] and calculating metabolic equivalent (MET) at baseline and the end of the study.

### Blood samples and biochemical assessments

A sample of 5 ml blood was collected from all participants after a 12 to 14 h fast, at baseline and at the end of week 15. These samples were centrifuged at 4000 rpm for 15 min. The samples of serum were separated into small aliquots and were frozen at − 80 °C. For Zn analysis, all tubes were washed by acid and rinsed with distilled water, then atomic absorption spectrometry (variant Chemthech Analytical 2000) was used to determine serum Zn concentration [39, 40]. Serum concentration of high-sensitivity C-reactive protein (hs-CRP) was determined by enzyme-linked immunosorbent assay (ELISA) kits (Diagnostics Biochem Canada, Ontario, Canada) with an intra-assay coefficient of variation (CV) of 7.2%. Serum TNF-α was measured by ELISA kits (Diaclone, Besancon, France). Intra-assay CV for serum TNF-αwas 6.5%. Serum apelin concentration was assessed by ELISA kits (ZellBio GmbH, Ulm, Germany), with an intra-assay CV of 7.2%. Serum insulin was determined by ELISA kits (Monobind, USA), with an intra-assay CV of 7.4%. Serum glucosewas measured by commercial kits (Pars Azemoon, Tehran, Iran) with the aid of a Selectra 2 Autoanalyzer (Vital Scientific, Spankeren, The Netherlands). Homeostatic Model Assessment of Insulin Resistance (HOMA-IR) was determined using the following equation:

$${\text{HOMA-IR}}\, = \,\left[ {{\text{Fasting serum glucose }}\left( {{\text{mg}}/{\text{dL}}} \right)\, \times \,{\text{Insulin }}\left( {\upmu{\text{U}}/{\text{L}}} \right)} \right]/405$$

### Statistical analysis

Intention-to-treat principle was applied for anthropometric and dietary intake variables. Per-protocol analysis (PPA) was performed for analyzing the biochemical data. Data analysis was performed using SPSS version 20. The results are presented as mean (± SD) and frequency (percent) for quantitative and qualitative variables, respectively. The Kolmogorov–Smirnov test was used to assess normal distribution of data. None normal data distribution has been presented as 25/75 IQR. Natural log transformations on plasma Zn, insulin, TNF-α, NPY, apelin and HOMA-IR were transformed through Box-Cox transformation. To compare qualitative variables between the two groups, the Chi square test was used. We used a *t* test and paired t-test to compare quantitative parameters between and within groups, respectively. In addition, because anthropometric parameters were measured 3 times during the study, analysis of variance for repeated measurements was used to compare data between various times. Analysis of covariance was performed in order to remove the effect of confounding factors. In this study, *P* values of less than 0.05 were considered statistically significant.

## Results

**Fig. 1:**
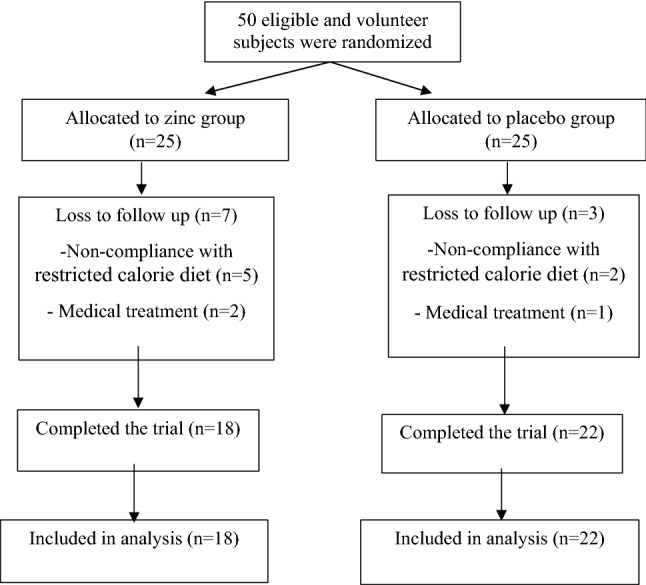
Flow diagram of the study

Of the 50 subjects initially enrolled, 10 subjects were eliminated because of non- compliance and medical treatment (Fig. 1). The baseline characteristics of the subjects did not differ significantly between the two groups (Table 1).

**Table 1:** Baseline characteristics of subjects in the Zinc and Placebo groups

Characteristics	Zinc (n = 18)	Placebo (n = 22)
Age (years)^a^	35.63 ± 3.2	32.95 ± 1.7
Sex (n/%)		
Male	6 (24%)	8 (32%)
Female	19 (76%)	17 (68%)
Past experiences with weight-reducing treatment (n/%)		
Yes	15 (60%)	13 (52%)
No	10 (40%)	12 (48%)
Marital status (n/%)		
Single	8 (32%)	9 (36%)
Married	17 (68%)	16 (64%)

^a^Values are mean ± SD

### Dietary intakes and physical activity

As is shown in Table 2, dietary intakes of energy, protein, carbohydrate, fat, saturated fatty acids (SAFA), monounsaturated fatty acids (MUFA), polyunsaturated fatty acids (PUFA), cholesterol, and Zn were not significantly different between the groups at baseline and the end of week 15. No significant changes were observed in physical activity levels between the two groups during the study.

**Table 2:** Dietary intakes and physical activity in the Zinc and Placebo groups

Variables	Zinc group			Placebo group			p-value		
	Baseline	Week 15	Mean change	Baseline	Week 15	Mean change	P^1^	P^2^	P^3^
Energy (kcal/d)	1727.12 ± 378.76	1463.49 ± 424.07	− 263.62 ± 514.36	1643.97 ± 476.19	1542.96 ± 500.29	− 101.01 ± 481. 08	0.498	0.547	0.254
P^4^	0.017			0.304					
Protein (g/d)	64.34 ± 11.42	60.64 ± 7.16	− 9.63 ± 6.98	69.73 ± 11.78	61.44 ± 19.38	− 7.52 ± 18.99	0.156	0.688	0.453
P^4^	0.209			0.057					
Carbohydrate (g/d)	237.99 ± 60.18	201.79 ± 65.89	− 36.27 ± 82.69	223.19 ± 55.26	208.57 ± 69.33	− 14.62 ± 57.37	0.370	0.722	0.288
P^4^	0.038			0.215					
Fat (g/d)	58.83 ± 15.43	50.64 ± 15.36	− 8.18 ± 22.28	55.31 ± 19.76	49.23 ± 15.93	− 6.07 ± 20.93	0.486	0.752	0.731
P^4^	0.079			0.160					
SFA (g/d)	14.01 ± 4.55	12.64 ± 3.91	− 1.37 ± 5.25	16.34 ± 15.06	14.84 ± 4.95	− 1.50 ± 14.45	0.483	0.089	0.965
P^4^	0.205			0.607					
MUFA (g/d)	18.41 ± 5.78	17.69 ± 5.48	− .71 ± 4.10	17.79 ± 6.52	18.98 ± 5.25	0.53 ± 8.00	0.722	0.683	0.491
P^4^	0.391			0.742					
PUFA (g/d)	23.70 ± 8.84	18.95 ± 8.68	− 4.75 ± 10.68	20.57 ± 8.92	16.23 ± 8.58	− 4.34 ± 10.04	0.219	0.272	0.88
P^4^	0.036			0.041					
Cholesterol (mg/d)	179.86 ± 87.60	184.76 ± 92.00	4.89 ± 110.06	189.21 ± 67.11	1999.92 ± 86.21	10.71 ± 77.50	0.674	0.550	0.830
P^4^	0.826			0.496					
Zinc (mg/d)	14.01 ± 1.14	14.02 ± 0.74	0.19 ± 0.94	13.5 ± 1.18	13.80 ± 1.34	0.30 ± 0.70	0.127	0.204	0.624
P^4^	0.317			0.04					
Physical activity (MET/day)	32.87 ± 3.40	32.77 ± 3.62	− 0.09 ± 0.67	32.23 ± 4.38	32.44 ± 4.33	0.21 ± 0.65	0.567	0.770	0.107
P^4^	0.481			0.119					

All values are mean ± SD

*SFA* saturated fatty acids, *MUFA* monounsaturated fatty acid, *PUFA* polyunsaturated fatty acid

P^1^: p-values for comparison of variables between two group by independent T-test at baseline

P^2^: p-values for comparison of variables between two group by independent T-test at week 15

P^3^: p-values for comparison of mean change of variables between two group by independent T-test

P^4^: p-values for comparison of variables within groups by Paired T-test

### Effects on anthropometric measurements

Weight, BMI, waist circumference and hip circumference decreased in both groups compared to baseline. However, the reductions of weight (P = 0.032), BMI (P = 0.025), waist circumference (P = 0.003) and hip circumference (P = 0.0001) were significantly higher in the Zn group than in the placebo group (Table 3). No significant change was observed in WHR within each group during the study (Table 3).

**Table 3:** Anthropometric parameters in Zinc and Control groups

Variable	Time	Zinc group	Placebo group	P^*^
Body weight (kg)	Baseline	89.59 ± 17.10	88.41 ± 12.46	0.781
	Week 7	83.16 ± 14.52	87.09 ± 12.53	0.312
	Week 15	84.99 ± 13.41	86.93 ± 12.34	0.597
	P**	0.020	0.007	
	Mean change^a^	− 4.60 ± 8.80	− 1.48 ± 2.37	0.093
	P^***^			0.032
BMI (kg/m^2^)	Baseline	33.17 ± 6.34	32.64 ± 2.37	0.701
	Week 7	30.66 ± 4.10	32.16 ± 2.63	0.129
	Week 15	31.50 ± 5.08	32.09 ± 2.31	0.599
	P**	0.024	0.007	
	Mean change	− 1.66 ± 3.33	− 0.55 ± .89	0.113
	P^***^			0.025
Waist circumference (cm)	Baseline	99.48 ± 10.19	99.32 ± 9.42	0.954
	Week 7	96.80 ± 10.07	98.10 ± 9.44	0.639
	Week 15	94.36 ± 10.31	97.82 ± 9.90	0.231
	P***	0.105	0.023	
	Mean change	− 5.12 ± 6.67	− 1.49 ± 3.52	0.020
	P^***^			0.003
Hip circumference (cm)	Baseline	114.72 ± 8.77	115.16 ± 5.49	0.833
	Week 7	111.83 ± 8.17	114.68 ± 5.64	0.159
	Week 15	109.84 ± 7.53	114.68 ± 5.68	0.013
	P**	0.0001	0.063	
	Mean change	− 4.88 ± 3.58	− 0.48 ± 1.04	0.0001
	P^***^			0.0001
WHR	Baseline	0.87 ± 0.09	0.86 ± 0.06	0.734
	Week 7	0.86 ± 0.07	0.85 ± 0.06	0.597
	Week 15	0.86 ± 0.11	0.85 ± 0.07	0.710
	P**	0.864	0.149	0.880
	Mean change	− 0.07 ± 0.053	− 0.009 ± 0.028	
	P^***^			0.682

All values are mean ± SD

^a^Mean change for the 15-week period

P*: p-values for comparison of variables between two group by independent T-test

P**: p-values for comparison of variables within groups by analysis of variance for repeated measurement

P***: p-values for comparison between mean changes of variables by Analysis of covariance (adjusted for age, mean change of calorie intake, mean change of zinc intake)

### Effects on biochemical markers and appetite

Serum zinc concentration increased significantly in the Zn group at the end of week 15 compared with baseline (P = 0.0001), whereas no significant change was observed in the placebo group. The increment of serum zinc concentration in the Zn group was significant in comparison with the placebo group (P = 0.002; Table 4). Serum hs-CRP reduced significantly in the Zn group at the end of week 15 in comparison with baseline (P = 0.0001), whereas no significant change was observed in the placebo group. The reduction of serum hs-CRP in the Zn group was significant in comparison with the placebo group (P = .0001; Table 4). Serum TNF-α concentration did not significantly change within each group during the study (Table 4). Serum apelin reduced significantly in the Zn group at the end of week 15 in comparison with baseline (P = 0.042), whereas it increased significantly in the placebo group (P = 0.001). The reduction of serum apelin in the Zn group was significant in comparison with the placebo group (P = 0.001; Table 4). Serum glucose (P = 0.046) and insulin (P = 0.002) reduced significantly in the Zn group at the end of week 15 in comparison with baseline. However, these reductions in the Zn group were not significant in comparison with the placebo. In addition, HOMA-IR decreased significantly in the Zn group at the end of week 15 in comparison with baseline (P = 0.0001), whereas no significant change was observed in the placebo group. The reduction of HOMA-IR in the Zn group was significant in comparison with the placebo group (P = .031; Table 4). Serum NPY decreased in the Zn group and this reduction was significant in comparison with the placebo group (Table 4; P = 0.048); however, after statistical adjustment for age and calorie intake, the reduction of NPY in the Zn group was not significant in comparison with the placebo group. Appetite score decreased significantly in the Zn group at the end of week 15 in comparison with baseline (P = 0.004), whereas no significant change was observed in the placebo group. The reduction of appetite score in the Zn group was significant in comparison with the placebo group (P = .001; Table 4).

**Table 4:** Biochemical markers and appetite in the Zinc and Placebo groups

Variables	Zinc group			Placebo group			p-value			
	Baseline	Week 15	Mean change	Baseline	Week 15	Mean change	P^2^	P^3^	P^4^	P^5^
Zinc (µg/dL)^1^	65.2 ± 5.9	75.4 ± 8.2	10.2 ± 6.8	71.15 ± 13.2	68.15 ± 10	− 3 ± 13.1	0.086	0.018	0.0001	0.002
P^1^	0.0001			0.296						
hs-CRP (mg/L)^1^	5.27 ± 2.93^b^	3.37 ± 2.24	− 1.89 ± 1.60	4.75 ± 2.28	3.98 ± 2.04	− 0.07 ± 1.8	0.124	0.064	0.0001	0.0001
P^1^	0.0001			0.60						
TNF-α (pg/ml) ^2^	32.41 (10.92, 708.48) ^a^	30.43 (11.79, 546.19)	− 1.98	26.07 (13.19, 67.47)	25.07 (9.62, 94.65)	− 1.0	0.170	0.238	0.293	0.723
P^1^	0.473			0.451						
Apelin (pg/ml)^2^	1568.20 (1119, 3282)	1245.13 (482, 2087)	− 323.07	1493.45 (505, 4467)	1683.32 (963, 3804)	189.87	0.805	0.017	0.002	0.001
P^1^	0.042			0.001						
FBS (mg/dL)^1^	86.83 ± 11.94	83.50 ± 7.36	− 3.33 ± 6.56	86.90 ± 9.93	86.90 ± 13.42	0.00 ± 6.00	0.983	0.343	0.102	0.088
P^1^	0.046			1.00						
Insulin (microU/L)^2^	5.91 (1.7,18.60)	4.05 (1,9.1)	− 1.86	5.07 (1.8, 22.6)	5.08 (1.7, 27.2)	0.01	0.135	0.33	0.019	0.073
P^1^	0.002			0.735						
HOMA-IR^2^	1.35 (0.37, 2.81)	0.83 (0.21, 2.27)	− 0.52	1.02 (0.41, 5.88)	1.08 (0.33, 9.27)	0.06	0.160	0.294	0.011	0.031
P^1^	0.0001			0.757						
NPY (ng/l)^2^	306.1 (178.8, 1540.5)	273.8 (170.1, 1022.8)	− 32.27	400.9 (198.9, 2239.2)	411.6 (180.6, 1900.9)	10.72	0.147	0.037	0.048	0.151
P^1^	0.114			0.243						
Appetite^1^	16.00 ± 2.11	13.77 ± 2.36	− 2.22 ± 2.81	15.50 ± 1.80	15.45 ± 1.59	− 0.04 ± 1.59	0.426	0.011	0.007	0.001
P^1^	0.004			0.892						

^a^Values are geometric mean (minimum, maximum)

^b^Values are mean ± SD

P^1^: p-values for comparison of variables within groups by Paired T-test

P^2^: p-values for comparison of variables between two groups by independent T-test at baseline

P^3^: p-values for comparison of variables between two group by independent T-test at week15

P^4^: p-values for comparison of mean change of variables between two group by independent T-test

P^5^: p-values for comparison between mean changes of variables by Analysis of covariance (adjusted for age, and mean change of calorie intake)

## Discussion

In our study, mean serum zinc in the Zn group (65.2 ± 5.9 µg/dL) was lower than normal range (70–120 µg/dL) at baseline [41]. At the end of week 15, mean serum zinc increased significantly in the Zn group (75.4 ± 8.2 µg/dL), whereas no significant change was observed in the placebo group. In the present study, weight, BMI, waist circumference and hip circumference decreased in both groups compared to baseline. However, the reductions of these anthropometric parameters were significantly higher in the Zn group than in the placebo group. To our knowledge, this is the first study to evaluate the co-administration of Zn supplement and RCD in individuals with obesity. In agreement with the present study, Payahoo et al. [16] showed that daily administration of 30 mg zinc gluconate for 1 month reduced body weight, BMI and waist circumferences in the healthy obese adults. It is documented that body weight management requires restricting energy intake, and increasing energy expenditure [42]. No significant changes were observed in physical activity levels between the two groups. In our study, although the difference in energy intake between the two groups was not statistically significant, the reduction of energy intake was higher in the Zn group than in the placebo group. Based on previous studies, it seems that improvement in Zn status could have beneficial effects on food intake regulation [43]. One of the suggested mechanisms may be related to the favorable effect of improvement in Zn status on leptin regulation for inhibiting eating behaviors through reduction in neuropeptide Y mRNA level [44]. Zn deficiency and obesity can lead to leptin resistance which may increase NPY levels in the hypothalamus of rodents and men [45]. Previous findings also report that Zn deficiency can cause a 50% increase in NPY levels [46], but despite the higher level of NPY in Zn deficient rats, their food intake is reduced because of NPY resistance [46, 47]. On the other hand, previous reports imply that Zn has an essential role in serotonin synthesis which stimulates satiety and reduce food intake [48]. Based on our knowledge, the functional role of zinc status in weight or appetite management of individuals with obesity has not been revealed. However, the role of lower plasma zinc level in inhibiting TSH secretion [49] and the involvement of zinc in the production, storage and release of insulin were also previously showed [50]. So it seems reasonable that zinc level may have an essential role in weight or appetite management of individuals with obesity. In our study, the baseline Zn level was below than the normal range (70–120 µg/dL) [51] in the Zn group; however, Zn levels turn to a normal status after the supplementation. In agreement with previous studies, serum NPY decreased in the Zn group and this reduction was significant in comparison with the placebo group. In addition, appetite score decreased significantly in the Zn group at the end of week 15 in comparison with baseline, and this reduction was significant in comparison with the placebo group. Welch et al. [52] also documented that NPY not only effect on food intake but also seems to be associated with macronutrient selection, such a way that increase carbohydrate intake. In agreement with Welch et al. study, carbohydrate and fat intakes were significantly reduced in the Zn group as compared to the placebo group in our study. In the present study, serum hs-CRP, an inflammatory marker, reduced significantly in the Zn group at the end of week 15 in comparison with baseline, and this reduction was significant in comparison with the placebo group. Inflammation is one of the main complications of obesity [53] and weight loss through dietary restriction may have a favorable effect on obesity-related inflammatory status [54]. Selvin et al. [55] suggested that a 1 kg weight loss through changes in diet and lifestyle will lead to a 0.13 mg/L reduction in serum CRP level. In our study, Serum TNF-α concentration did not significantly change in the Zn group. In agreement with this study, Kim et al. [30] did not find any significant reduction in serum TNF-α after a 8-week supplementation with Zn. In addition, serum apelin, an adipose tissue inflammatory biomarker [28], reduced significantly in the Zn group at the end of week 15 in comparison with baseline, and this reduction was significant in comparison with the placebo group. To our knowledge, no studies to date have evaluated the effects of Zn supplementation on apelin levels; however, some studies revealed that weight loss with RCD can cause a significant reduction in apelin level [28, 56] which seems this reduction has been largely attributed to decreased inflammation or increased insulin sensitivity [28, 55–62]. Serum glucose and HOMA-IR reduced significantly in the Zn group at the end of week 15 in comparison with baseline. Insulin sensitivity improvement is documented in previous weight loss interventions using calorie restriction [57–59]. It has been shown that a 5–10% weight loss increases insulin sensitivity [60, 61]. However, the effectiveness of the Zn supplementation on IR is controversial [18, 62, 63]. It seems that zinc supplementation with longer duration has more favorable effects on IR or glucose tolerance [64, 65]. One of the probable mechanisms for the beneficial effects of Zn on IR may be related to decreased inflammation [66]. Few studies have proposed that higher levels of hs-CRP are associated with insulin resistance and hyperinsulinemia [67–69]. Furthermore, the role of apelin in the development of insulin resistance has also attracted a lot of attention in the recent years [70, 71]. It has been shown that apelin level is higher in insulin-resistant individuals and it has also been suggested that apelin can inhibit the insulin secretion [70, 72, 73]. The proposed mechanisms for the role of apelin in insulin sensitivity include direct effects on glucose uptake or insulin signaling pathways and indirect effects on energy metabolism [28]. A limitation of our study was the small sample size.

## Conclusion

This study indicates that Zn supplementation with a restricted calorie diet has favorable effects in reducing anthropometric measurements, inflammatory markers, insulin resistance and appetite in individuals with obesity, and may play an effective role in the treatment of obesity.

## Data Availability

The datasets used and analyzed during the current study are available from the corresponding author on reasonable request.

## Abbreviations

Zn: zinc
RCD: restricted calorie diet
HOMA-IR: homeostatic model assessment of insulin resistance
hs-CRP: high-sensitivity C-reactive protein
BMI: body mass index
IR: insulin resistance
IL-6: interleukin-6
TNF-α: tumor necrosis factor-α
NPY: neuropeptide Y
SNAQ: Simplified Nutritional Appetite Questionnaire
MET: metabolic equivalent
PPA: per-protocol analysis
SAFA: saturated fatty acids
MUFA: monounsaturated fatty acids
PUFA: polyunsaturated fatty acids
